# Effects of Combined Application of Biochar and Nitrogen Fertilizer on Forage Growth and Water- and Nitrogen-Use Efficiency in Managed Grassland

**DOI:** 10.3390/plants15142203

**Published:** 2026-07-19

**Authors:** Rong Zhang, Die Chen, Die Lu, Helong Yang, Jiuqi Zhao, Wenshan Chen, Mengyue Wang, Kejian Lin, Zhi Xing, Lingqi Kong, Qianwei Guo, Boyan Wang

**Affiliations:** 1 College of Grassland Science, Xinjiang Agricultural University, Urumqi 830052, China; 18085849247@163.com (D.C.); iludieya@163.com (D.L.); xndzjq@163.com (J.Z.); 15863671865@163.com (W.C.); 15035964969@163.com (M.W.); 2 Xinjiang Key Laboratory of Grassland Resources and Ecology, Urumqi 830052, China; 3 Key Laboratory of Grassland Resources and Ecology of Western Arid Region, Ministry of Education, Urumqi 830052, China; 4 Institute of Grassland Research, Chinese Academy of Agricultural Sciences, Hohhot 010010, China; linkejian@caas.cn (K.L.); xingzhi@caas.cn (Z.X.); konglingqi@caas.cn (L.K.); 5 Grassland Workstation of Chahar Right Front Banner, Ulanqab 012200, China; guoqianwei0921@126.com; 6 Baotou Steel Group Design and Research Institute, Baotou 014000, China; 18947720217@163.com

**Keywords:** modeling biochar, nitrogen fertilizer, improved grassland, water and nitrogen dynamics, water- and nitrogen-use efficiency

## Abstract

To tackle the low nitrogen-use efficiency, water scarcity, and insufficient forage supply in improved grasslands in arid Northwest China, a two-factor field trial involving three biochar dosages (0, 10, 20 t ha^−1^) and two nitrogen (N) application rates (0, 225 kg ha^−1^) was conducted on mountain grassland in Xinjiang, with *Onobrychis viciifolia* and *Bromus inermis* as test forages. Soil physicochemical traits, soil–forage and water–nitrogen dynamics, forage productivity, and economic returns were determined, and partial least squares path modeling (PLS-PM, GoF = 0.373) was conducted to quantify the regulatory pathways. Co-treatments with biochar and N markedly lowered soil bulk density and elevated porosity, soil organic carbon, and the available N pool; application of biochar deferred the peak of soil available N and prolonged fertilizer efficacy by more than seven days. Forage total nitrogen- and water-use efficiency peaked at the first cutting, with the B1N1 treatment (10 t ha^−1^ biochar + 225 kg ha^−1^ N) boosting water-use efficiency by 45.2%. *O. viciifolia* exhibited optimal performance under B1N15, while B2N15 (20 t ha^−1^ biochar + 225 kg ha^−1^ N) was more favorable for *B. inermis*. The B1N15 regime achieved the highest net profit of CNY 12,747.99 ha^−1^, but high biochar input cut economic gains by 14.1%. The PLS-PM model uncovered a dual regulatory mechanism: combined amendments directly optimized soil microhabitats, and soil water–nitrogen conditions served as a central mediator to facilitate biomass accumulation and economic gains through cascading soil–vegetation transmission, without direct impacts on vegetation productivity or economic outputs. This research provides a scientific reference for the use of fertilization in the sustainable management of arid improved grasslands.

## 1. Introduction

As an essential agricultural fertilizer, nitrogen fertilizer plays a pivotal role in agricultural production. Currently, the average utilization efficiency of nitrogen fertilizer worldwide is less than 50% [1]. The unused nitrogen fertilizer is partially lost via surface runoff and soil leaching into soil and groundwater, triggering ecological problems such as water eutrophication. The rest is emitted into the atmosphere in the form of nitrous oxide, further exacerbating global warming. In recent years, with the continuous intensification of climate change, extreme weather events, including drought, flood, and heavy rainfall, have occurred more frequently. In particular, drought has threatened global agricultural production, seriously restricting the development of the ecological environment and agricultural industries [2]. Drought exerts a strong influence on forage growth as well as water and nitrogen utilization; however, soil amendments can be applied to improve water- and nitrogen-use efficiency, enhance plant drought resistance, and alleviate the adverse effects of drought stress on plants.

Biochar is produced from biomass materials, such as crop straw and livestock manure, through high-temperature oxygen-free pyrolysis, and has attracted worldwide attention as a carbon sequestration and soil-amendment material [3]. It can not only improve soil fertility and crop yield, but also optimize crop quality [4]. Biochar possesses an average specific surface area of up to 125 m^2^/g, with abundant internal microporous structures, which can effectively increase soil capillary water-holding capacity after soil incorporation. Meanwhile, its surface is rich in hydrophilic functional groups, including carboxyl and hydroxyl groups [5], endowing it with strong water adsorption and water retention capacity. The application of biochar can reduce soil bulk density and soil acidity, raise fertilizer-use efficiency, and strengthen soil water and nutrient retention capacity [6]. Combined application of biochar with conventional fertilizers can dramatically improve soil physicochemical properties and reduce fertilizer waste. Existing mechanistic studies have confirmed that the porous structure, large specific surface area, and surface hydrophilic functional groups of biochar can mediate the transformation and retention process of soil inorganic nitrogen, which is the core internal link between biochar application and nitrogen fertilizer-use efficiency [3,5,6]. It can immobilize ammonium nitrogen through electrostatic adsorption and intercept nitrate nitrogen in pores to reduce nitrogen leaching and gaseous loss; meanwhile, it optimizes soil hydrothermal and aeration conditions to regulate the activity of nitrogen-transforming microorganisms, thereby synchronously adjusting the mineralization and immobilization balance of soil nitrogen and prolonging the effective supply period of nitrogen nutrients. However, the relationships between the above nitrogen retention mechanisms of biochar and the growth and nitrogen uptake characteristics of leguminous and gramineous forages under arid grassland conditions still require further systematic verification. 

From the perspective of domestic agricultural production, nitrogen fertilizer is irreplaceable. Statistics show that the nitrogen fertilizer-use efficiency in China is less than 45%, which is lower than the global average [7]. Moreover, droughts occur frequently in many regions of China, such as Northwest China and the Huang He Plain. Located by the northwestern border of China, Xinjiang is among the regions most severely affected by drought [8]. Therefore, improving water- and nitrogen-use efficiency and reducing their loss have become urgent core issues restricting agricultural development in China.

As one of China’s major pastoral areas, Xinjiang boasts a total grassland area of 52 million hectares (including the XPCC), ranking third nationwide, with an overall grassland vegetation coverage of 41.6% according to official data. Nevertheless, affected by natural and human factors, grassland degradation is severe, resulting in a shrinking area and declining productivity; natural grasslands can no longer satisfy the demands of animal husbandry development. Accordingly, the development focus of animal husbandry in Xinjiang is gradually shifting from traditional grassland grazing to indoor feeding in agricultural regions. Insufficient forage supply has long been the key bottleneck restricting the modernization and large-scale development of local animal husbandry [8,9].

The development of improved grassland serves as a vital approach in alleviating the shortage of forage supply from natural grasslands and enhancing grassland productivity. Combined with the biological characteristics of forage species and regional climatic conditions, targeted grassland communities can be established via scientific agronomic practices such as sowing and fertilization. These practices effectively offset the productivity decline caused by natural grassland degradation and provide solid support for the sustainable development of animal husbandry [10].

Sainfoin (*Onobrychis viciifolia*) and smooth bromegrass (*Bromus inermis*) are the main high-quality forage species used for improved grassland establishment in Northwest China. Sainfoin has a high yield and abundant tannins, which can prevent livestock bloat disease, and is also rich in protein and amino acids; hence, it is known as the “Queen of Forages” [11]. As a perennial gramineous forage, smooth brome features well-developed rhizomes and a high biomass yield, making it a preferred grass species for soil and water conservation as well as grain-forage conversion projects [12].

In this study, improved grassland was selected as the research object. On the basis of conventional nitrogen application, different supplementary biochar application rates were used. Based on the existing research findings of biochar-mediated nitrogen retention, this study puts forward two testable hypotheses: (1) co-application of biochar and nitrogen fertilizer can modify soil physical and chemical microhabitats, optimize the spatiotemporal dynamic distribution of soil water and available nitrogen, and generate species-specific differences in nitrogen absorption and utilization between *O. viciifolia* and *B. inermis*; and (2) the combined amendments regulate the comprehensive benefits of improved grassland through a two-stage cascade transmission system centered on soil water–nitrogen dynamics, while exogenous biochar and nitrogen treatments have no direct driving effects on forage biomass and economic benefits. 

To further clarify the internal relationships between soil environment, vegetation growth, and economic benefits under combined biochar and nitrogen application, the partial least squares path model (PLS-PM) was adopted. A causal network of “regulatory factors–soil environment–vegetation growth–economic benefits” was established to quantify the direct and indirect effect intensity among various variables. The objective of this study was to understand the ability of biochar to improve soil physicochemical properties, enhance drought-resistant physiological characteristics of forages, and raise resource-use efficiency under arid conditions.

## 2. Results

### 2.1. Effects of Combined Application of Biochar and Nitrogen Fertilizer on Soil and Forage Water and Nitrogen Dynamics in Improved Grassland

The combined application of biochar and nitrogen fertilizer markedly improved soil physical structure and chemical fertility in improved grassland, regulated the temporal and spatial dynamics of soil water and nitrogen, prolonged nitrogen fertilizer efficiency, and effectively increased nitrogen accumulation in forages. 

**Figure 1:**
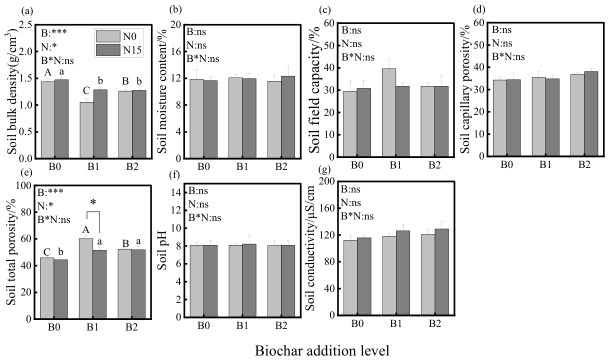
Effects of different fertilization methods on soil physical properties of improved grassland. (**a**) Soil bulk density; (**b**) Soil moisture; (**c**) Field capacity; (**d**) Capillary porosity; (**e**) Total porosity; (**f**) Soil pH; (**g**) Electrical conductivity under different fertilization treatments, Data is expressed as mean ± standard error. The application rates of nitrogen fertilizer are N0: 0 kg/ha and N15: 225 kg/ha. The application rates of biochar are B0: 0 t/ha, B1:10 t/ha, and B2: 20 t/ha. ns: not significant. Different uppercase letters indicate significant differences (*p* < 0.05) between different biomass charcoal treatments under the level of no nitrogen fertilizer (N0) applied, while different lowercase letters indicate significant differences (*p* < 0.05) between different biomass charcoal treatments under the level of conventional nitrogen fertilizer (N15) applied. *, *** indicate significant differences in nitrogen fertilizer application under the same biochar treatment (*p* < 0.05).

In terms of soil physical properties, the combined application greatly reduced soil bulk density and increased total soil porosity. Under a nitrogen application rate of 225 kg/hm^2^, the treatments with 10 t/hm^2^ and 20 t/hm^2^ of biochar decreased the soil bulk density by 12.3% and 18.7%, and increased total porosity by 15.6% and 22.4%, respectively, compared with the biochar-free group, which greatly improved soil looseness (Figure 1). There were no significant differences in soil field capacity between any of the biochar and nitrogen fertilization treatments (Figure 1c).

For soil chemical properties, co-application significantly elevated the contents of soil organic carbon, total nitrogen, ammonium nitrogen, nitrate nitrogen, and available nitrogen. The 20 t/hm^2^ biochar treatment combined with nitrogen fertilizer increased soil organic carbon by 37.7% and total nitrogen by 19.5% relative to the control, providing a stable nutrient supply for forage growth (Figures S1 and S2).

**Figure 2:**
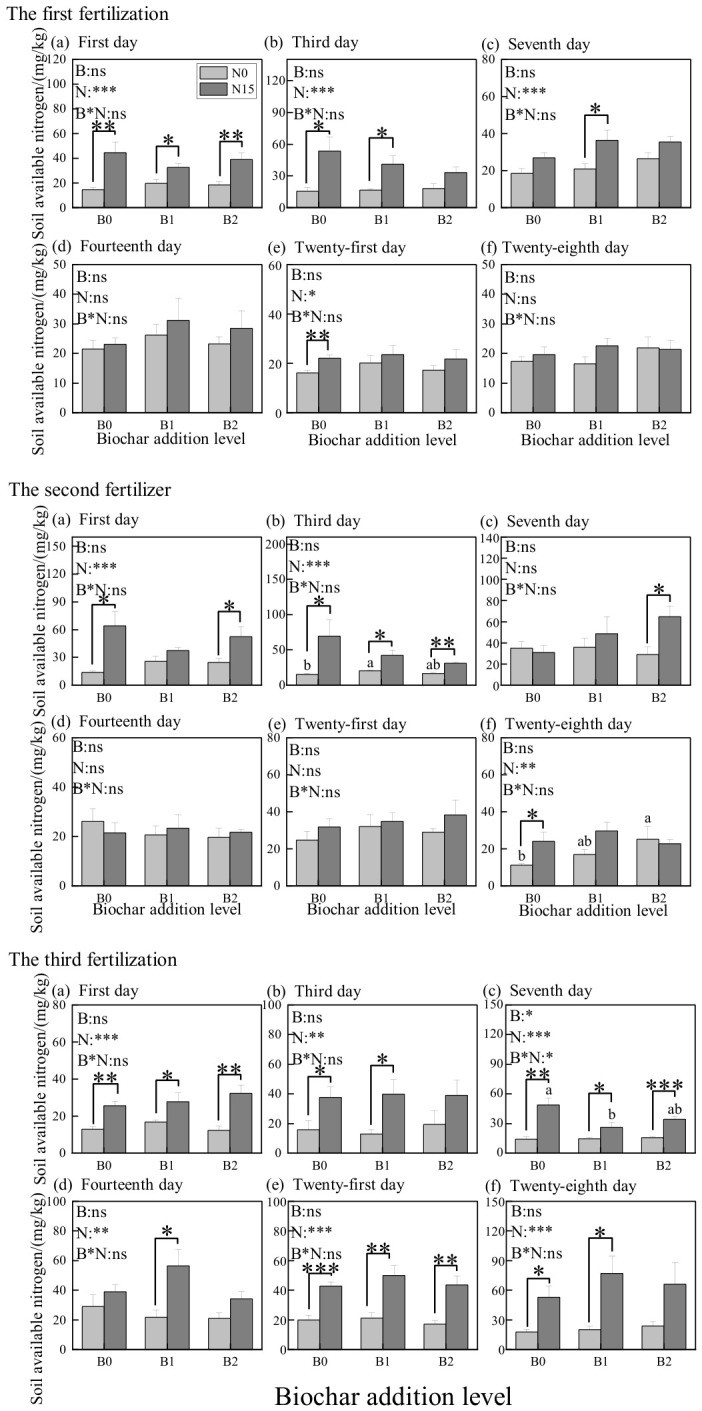
Effects of different fertilization methods on dynamic changes of soil available nitrogen in improved grassland after the third fertilization. Data is expressed as mean ± standard error. The application rates of nitrogen fertilizer are N0: 0 kg/ha and N15: 225 kg/ha. The application rates of biochar are B0:0 t/ha, B1: 10 t/ha, and B2: 20 t/ha. ns: not significant. While different lowercase letters indicate significant differences (*p* < 0.05) between different biomass charcoal treatments under the level of conventional nitrogen fertilizer (N15) applied. *, **, *** indicate significant differences in nitrogen fertilizer application under the same biochar treatment (*p* < 0.05, *p* < 0.01, *p* < 0.001); the data are derived from combined, randomly collected soil samples from the plots.

Soil water and nitrogen dynamic monitoring revealed that biochar exerted prominent water retention effects, with the most obvious improvement in soil water content observed in September (Figures S3 and S4). Meanwhile, biochar showed a mild delaying effect on soil available nitrogen peaks; this effect was inconsistent across three fertilization cycles. Soil nitrate nitrogen and available nitrogen in treatments without biochar peaked within 1–3 days after fertilization and then declined rapidly, whereas biochar-augmented treatments maintained a high nitrogen supply level until 7 days after fertilization. Biochar also significantly enhanced ammonium nitrogen retention capacity, extending nitrogen fertilizer efficiency by more than 7 days (Figure 2, Figures S5 and S6). Significant B × N interactive effects were only found on day 7 of the third fertilization, with no significant interaction observed at any other sampling time.

**Figure 3:**
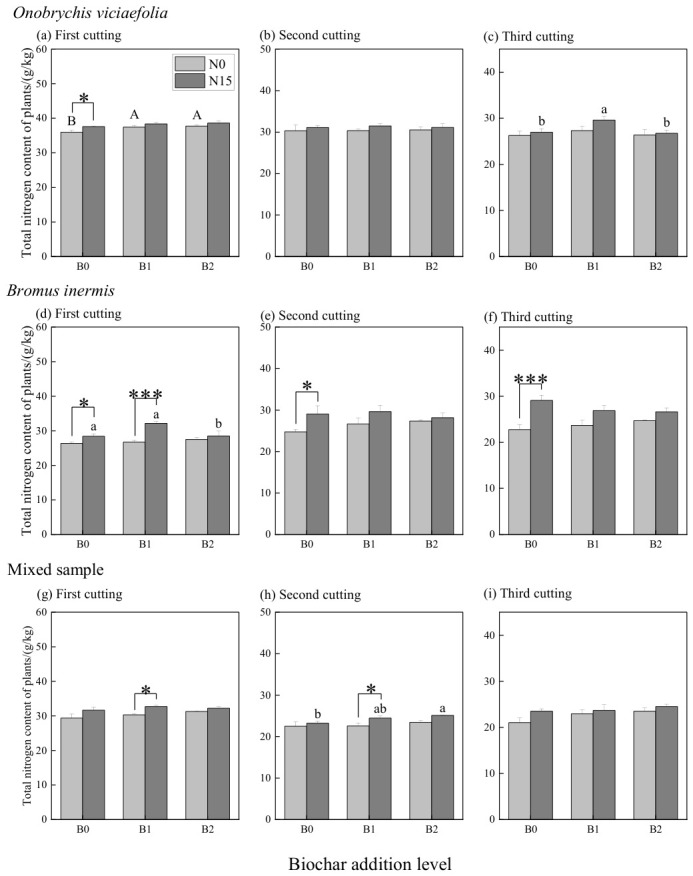
Effects of different fertilization methods on the total nitrogen content of forage. Data is expressed as mean ± standard error. The application rates of nitrogen fertilizer are N0: 0 kg/ha and N15: 225 kg/ha. The application rates of biochar are B0: 0 t/ha, B1: 10 t/ha, and B2: 20 t/ha. Different uppercase letters indicate significant differences (*p* < 0.05) between different biomass charcoal treatments under the level of no nitrogen fertilizer (N0) applied, while different lowercase letters indicate significant differences (*p* < 0.05) between different biomass charcoal treatments under the level of conventional nitrogen fertilizer (N15) applied. *, *** indicate significant differences in nitrogen fertilizer application under the same biochar treatment (*p* < 0.05, *p* < 0.001).

In terms of forage responses, combined fertilization notably increased the leaf total nitrogen content of *O. viciifolia* and *B. inermis*, showing an obvious trend of the first cut > the second cut > the third cut across different cutting stages. Compared with the control, the total nitrogen content of the first-cut *O. viciifolia* increased by 19.5% under 10 t/hm^2^ biochar combined with nitrogen fertilizer, and that of *B. inermis* increased by 23.2% under 20 t/hm^2^ biochar combined with nitrogen fertilizer. The annual nitrogen accumulation of the mixed-sown grassland community increased by over 30% (Figure 3, Figures S7 and S8).

### 2.2. Effects of Combined Biochar and Nitrogen Fertilizer Application on Water- and Nitrogen-Use Efficiency and Economic Benefits of Improved Grassland

The combined application of biochar and nitrogen fertilizer significantly increased the water-use efficiency at the first cutting, the nitrogen fertilizer-use efficiency, and the fresh forage yield in improved grassland, while its economic benefit showed a threshold effect as the biochar application rate increased.

**Figure 4:**
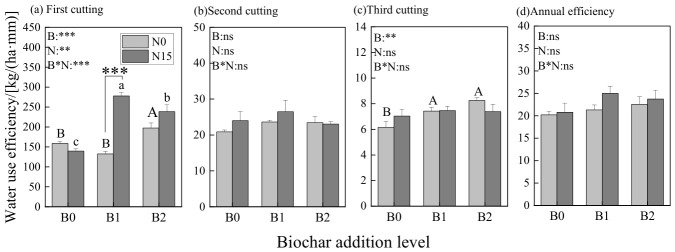
Effects of different fertilization methods on water-use efficiency of improved grassland. Data is expressed as mean ± standard error. The application rates of nitrogen fertilizer are N0: 0 kg/ha and N15: 225 kg/ha. ns: not significant. The application rates of biochar are B0: 0 t/ha, B1: 10 t/ha, and B2: 20 t/ha. Different uppercase letters indicate significant differences (*p* < 0.05) between different biomass charcoal treatments under the level of no nitrogen fertilizer (N0) applied, while different lowercase letters indicate significant differences (*p* < 0.05) between different biomass charcoal treatments under the level of conventional nitrogen fertilizer (N15) applied. *, **, *** indicate significant differences in nitrogen fertilizer application under the same biochar treatment (*p* < 0.01, *p* < 0.001).

In terms of water use efficiency (WUE), combined fertilization markedly improved WUE at the first cutting, while biochar application significantly elevated WUE at the third cutting. Relative to the control, co-application of nitrogen fertilizer with biochar at rates of 10 t/hm^2^ and 20 t/hm^2^ increased the first-cutting WUE by 45.2% and 52.7%, respectively (Figure 4).

**Figure 5:**
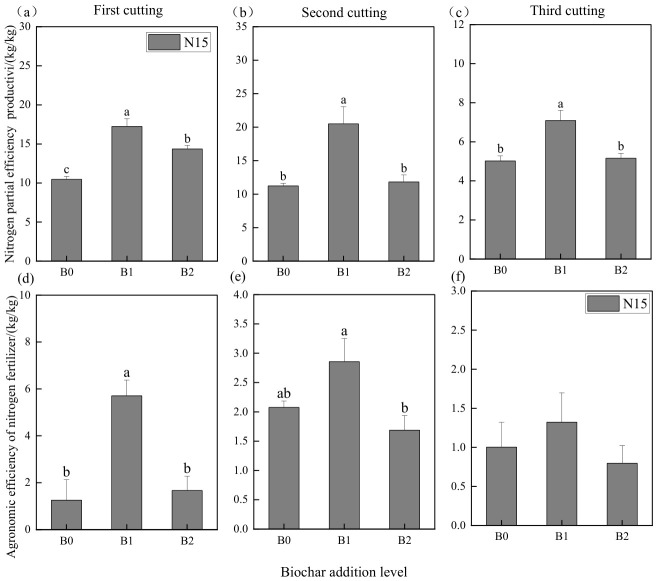
Effects of different fertilization strategies on nitrogen partial factor productivity and nitrogen agronomic efficiency in *O. viciifolia*. (**a**)Nitrogen partial productivity of the first cutting; (**b**)the second cutting and (**c**)the third cutting; (**d**)Agronomic efficiency of nitrogen fertilizer of the first cutting; (**e**)the second cutting and (**f**)the third cutting; Data is expressed as mean ± standard error. The application rates of nitrogen fertilizer are N0: 0 kg/ha and N15: 225 kg/ha. The application rates of biochar are B0: 0 t/ha, B1: 10 t/ha, and B2: 20 t/ha. Different letters indicate significant differences (*p* < 0.05) between different biomass charcoal treatments under the level of no nitrogen fertilizer (N0) applied, while different lowercase letters indicate significant differences (*p* < 0.05) between different biomass charcoal treatments under the level of conventional nitrogen fertilizer (N15) applied.

There were obvious species-specific differences in nitrogen fertilizer-use efficiency. *O. viciifolia* achieved the highest partial factor productivity and agronomic efficiency of nitrogen fertilizer use under 10 t/hm^2^ biochar combined with nitrogen fertilizer, while *B. inermis* exhibited the optimal nitrogen uptake efficiency under 20 t/hm^2^ biochar combined with nitrogen fertilizer. The nitrogen fertilizer-use efficiency ranked as follows across all harvest stages: the first cut > the second cut > the third cut. Nitrogen-use efficiency presented a declining trend under combined application, indicating that soil nitrogen was gradually enriched while its transformation efficiency tended to be stable (Figure 5 and Figures S9–S12).

**Figure 6:**
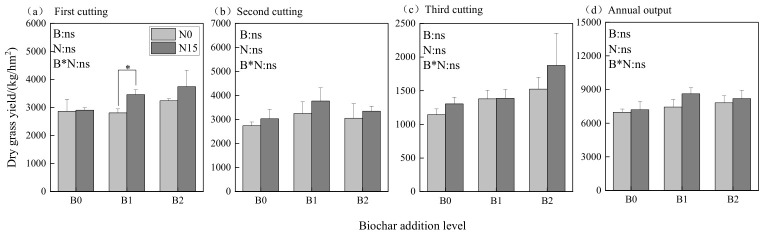
Effects of different fertilization methods on grass yield in improved grassland. Data is expressed as mean ± standard error. The application rates of nitrogen fertilizer are N0: 0 kg/ha and N15: 225 kg/ha. The application rates of biochar are B0: 0 t/ha, B1: 10 t/ha, and B2: 20 t/ha. ns: not significant. While different lowercase letters indicate significant differences (*p* < 0.05) between different biomass charcoal treatments under the level of conventional nitrogen fertilizer (N15) applied. * indicate significant differences in nitrogen fertilizer application under the same biochar treatment (*p* < 0.05).

The application of both biochar and nitrogen fertilizer increased the dry matter yield of forage, but their effects varied across cutting times and annual total dry matter yield. For the dry matter yield of the first cutting, nitrogen addition significantly elevated forage yield under the B1 biochar treatment (*p* < 0.05); biochar application exhibited a mild promoting effect without statistical significance. Single factors of biochar, nitrogen fertilizer, and their interaction exerted non-significant effects on the dry matter yield of the second cutting, third cutting, and annual total yield, although numerical yield improvements were observed for all treatments (Figure 6).

An economic benefit analysis showed that the 10 t/hm^2^ biochar treatment combined with nitrogen fertilizer achieved the highest net profit of CNY 12,747.99 per hectare. Due to the excessive input cost of biochar, the net profit when using 20 t/hm^2^ biochar combined with nitrogen fertilizer decreased by 14.1% compared with the optimal treatment (Table 1).

A comprehensive evaluation based on membership function, considering the soil improvement effect, forage yield, water- and nitrogen-use efficiency, and economic benefits revealed that the combination of 10 t/hm^2^ biochar and 225 kg/hm^2^ nitrogen fertilizer was the optimal formula for balancing production performance and economic returns. By contrast, the application of 20 t/hm^2^ biochar combined with nitrogen fertilizer was more suitable for grassland management aimed at long-term soil amelioration (Table S1; Figure S13).

### 2.3. Analysis of Action Pathways Linking Soil, Vegetation, and Economic Benefits

**Figure 7:**
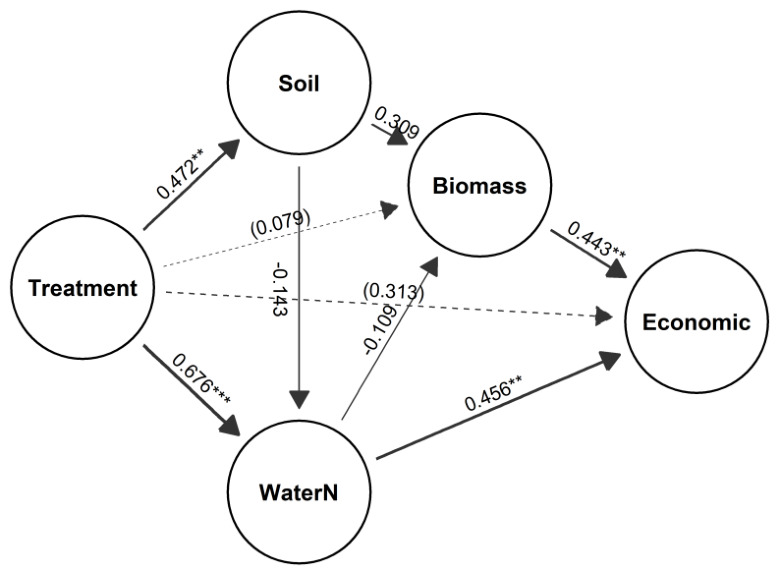
Partial least squares path model for regulating productivity and economic benefits of improved grassland via combined application of biochar and nitrogen fertilizer. Solid lines represent direct effects, dashed lines represent indirect effects; values are standardized path coefficients. **: *p* < 0.01, ***: *p* < 0.001.

Structural equation modeling (SEM; GoF = 0.373) was used to quantify the direct and indirect pathways linking fertilization, soil properties, vegetation biomass, and economic benefits (Figure 7). A path analysis revealed that exogenous fertilization treatments had no direct driving effect on vegetation biomass or economic benefits; instead, treatments improved economic outcomes entirely through indirect pathways. Specifically, fertilization significantly enhanced soil physicochemical properties (0.472, *p* < 0.01) and water–nitrogen dynamics (0.676, *p* < 0.001); then, the enhanced water–nitrogen dynamics (0.456, *p* < 0.01) and the subsequent increases in vegetation biomass (0.443, *p* < 0.01) directly and positively determined the final economic benefits of the ecosystem.

## 3. Discussion

### 3.1. Module 1: Integrated Analysis of Biochar-Mediated Soil Physicochemical Improvement and Nitrogen Cycling Regulation Mechanisms

Combined application of biochar and nitrogen fertilizer profoundly reshaped the physicochemical properties of improved grassland and drove sequential changes in soil nitrogen transformation and forage nitrogen uptake; all relevant mechanistic evidence in this study aligns with the conclusions of previous research [13]. The field measurement data (Figure 1) demonstrated that biochar’s porous architecture and large specific surface area filled inter-particle gaps and reduced soil cohesion and compaction; under the 225 kg/ha nitrogen application, the 10 t/ha and 20 t/ha biochar treatments decreased soil bulk density by 12.3% and 18.7%, and increased total soil porosity by 15.6% and 18.7%, respectively [5,6]; meanwhile, exogenous nitrogen input stimulated the proliferation of grass root systems, and root penetration further loosened topsoil to form a synergistic physical amelioration effect together with the biochar amendments [14]. No significant inter-treatment difference was observed for field water capacity (Figure 1c). Chemically, co-application significantly raised the soil organic carbon and total nitrogen concentrations: the 20 t/ha biochar-plus-nitrogen treatment increased soil organic carbon by 37.7% and total nitrogen by 19.5% relative to the control group. Biochar acts as a stable exogenous carbon source to directly supplement the soil organic carbon pool, and the improvements in soil aeration and moisture conditions that it causes elevate soil microbial activity to accelerate plant residue mineralization and indirectly accumulate soil organic matter, while the applied nitrogen supplies sufficient substrate for microbial metabolism to facilitate such carbon sequestration processes [3,15]. Such simultaneous optimization of the soil physical and chemical environments laid the foundation for the retention and slow release of soil nitrogen nutrients [16], and the whole series of nitrogen regulation processes triggered by biochar can be systematically interpreted from physical adsorption, chemical immobilization, and microbial modulation perspectives [5,6,17]. Dynamic monitoring of soil available nitrogen (Figure 2) validated the nitrogen-retention function of biochar: the single nitrogen treatments peaked in nitrate and available nitrogen within 1–3 days after fertilization and then declined rapidly, while the biochar-augmented groups maintained high available nitrogen levels until day 7 after fertilization, extending nitrogen fertilizer efficacy for more than seven days, though this delay was inconsistent across multiple fertilization cycles [18,19]. Biochar’s negatively charged surface functional groups immobilized ammonium nitrogen via electrostatic adsorption to cut ammonia volatilization, and its porous structures intercepted nitrate nitrogen to mitigate leaching risks [20,21]; it also optimized soil water, pH, and aeration microhabitats to support nitrogen-fixing microbes and boost biological nitrogen fixation [17]. Biochar-mediated adjustment of the soil C/N ratio balanced microbial nitrogen immobilization and mineralization and stabilized the long-term inorganic nitrogen supply rhythm [22]. This steady nitrogen supply altered the nitrogen accumulation of *O. viciifolia* and *B. inermis* (Figure 3): the leaf total nitrogen of both forages followed the order: the first cut > the second cut > the third cut, across all mowing rounds. Compared with the control, the 10 t/ha biochar-plus-nitrogen treatment increased the first-cut total nitrogen of sainfoin by 19.5%, and the 20 t/ha biochar-plus-nitrogen treatment raised the smooth brome first-cut nitrogen by 23.2%; the annual nitrogen accumulation of mixed-grassland communities rose by over 30%. The sequential decline in forage nitrogen with successive mowing can be explained by the gradual depletion of soil nitrogen reserves and weakened photosynthetic metabolism in late growth periods [3,23,24]. Obvious species-specific differences in nitrogen utilization were observed: sainfoin achieved maximum nitrogen accumulation under B1N15, while smooth brome showed optimal nitrogen uptake under B2N15 (Figure 5), which was the result of divergent root traits and differences in nitrogen preference between legume and grass species [25]. The improved soil water retention effected by biochar constituted a precondition for efficient nitrogen absorption; biochar’s hydrophilic functional groups enhanced soil water storage and reduced evaporation and surface runoff, while maintaining an adequate nitrogen supply, which promoted root growth and strengthened water uptake capacity [26,27,28,29,30]. The synergism between biochar’s water retention and slow nitrogen release guaranteed a matched water–nitrogen supply throughout the whole growth season, serving as the core driver of elevated soil–forage nitrogen transformation efficiency; the coupled physical–chemical–biological functions were not simply superimposed on each other, but formed integrated synergistic feedback loops to continuously replenish the soil nutrient pools and sustain stable nitrogen assimilation by forages [31,32].

### 3.2. Module 2: Comprehensive Coupling of Water- and Nitrogen-Use Efficiency, Forage Productivity, and Ecosystem Multi-Path Regulation

The optimized soil water and nitrogen microenvironment induced by biochar and nitrogen co-treatments further shaped variations in water-use efficiency, forage yield, and economic returns, with PLS-PM (Figure 7, GoF = 0.373) quantitatively clarifying the cascading transmission between exogenous fertilization, soil conditions, and ecosystem benefits [33,34]. A significant improvement in water-use efficiency only occurred in the first and third mowing stages (Figure 4): the 10 t/ha and 20 t/ha biochar-plus-nitrogen treatments increased first-cut water-use efficiency by 45.2% and 52.7%, respectively, with no significant differences detected at the second cut or in the annual average water-use efficiency; the soil water storage capacity exhibited no remarkable difference for any of the treatments. The improvement in water utilization was mainly caused by the matching of biochar’s slow-release nitrogen and the seasonal forage water demand, which maximized the utilization of stored soil water and reduced resource waste [35]. Consistent with species-specific nitrogen uptake, the nitrogen partial factor productivity and agronomic efficiency of sainfoin reached a peak under B1N15 (Figure 5), while smooth brome obtained optimal nitrogen uptake efficiency under B2N15. Nitrogen-use efficiency decreased gradually with mowing time [36,37]. Combined fertilization greatly elevated the fresh grass yield of the first cut and the annual total yield (Figure 6), with a decreasing yield trend through the second and third cuts, which matched the trend in nitrogen utilization across harvests [38,39]. The single-factor and interactive effects of biochar and nitrogen exerted no significant influence on the dry matter yield of the second and third cuts and the annual total yield, despite numerical increases. The economic analysis (Table 1) presented an obvious dosage threshold effect: the B1N15 treatment reached the highest net profit of CNY 12,747.99/ha, whereas B2N15 reduced the net profit by 14.1% due to the cost of biochar input. Multi-index membership function evaluation (Table S1) indicated that B1N15 balanced forage productivity and economic returns, while B2N15 was more suitable for long-term soil amelioration targets. The path modeling results (Figure 7) showed that combined biochar–nitrogen treatments exerted significant positive direct effects on soil physicochemical properties (0.472, *p* < 0.01) and soil water–nitrogen dynamics (0.676, *p* < 0.001), but there were no direct pathways affecting vegetation biomass or economic benefits. Soil water–nitrogen dynamics acted as the core mediator and generated significant positive direct effects on vegetation biomass (0.443, *p* < 0.01) and economic benefits (0.456, *p* < 0.01); exogenous fertilization produced indirect promotion effects of 0.079 and 0.313 on biomass and economic benefits, respectively, via soil cascading transmission [6,40,41,42]. This hierarchical transmission agreed with the general ecosystem resource–vegetation–benefit theory [38,43]. The model had higher explanatory power for soil water–nitrogen dynamics (R^2^ = 0.387) and economic benefits (R^2^ = 0.382), with lower R^2^ values for soil physicochemical properties and vegetation biomass, which could be attributed to unmodeled interference factors, including climate, interspecies competition, and mowing frequency [12,24]. In general, exogenous biochar and nitrogen inputs remodel soil water and nutrient habitats first, then sequentially promote forage biomass accumulation and economic profit growth through continuous soil–water–nitrogen chain conduction, forming a complete cascading regulatory loop within arid improved grassland ecosystems [44].

## 4. Materials and Methods

### 4.1. Study Area

The experiment was carried out at Xiejiagou Grassland Experimental Station of Xinjiang Agricultural University in Urumqi County, Xinjiang (43°52′ N, 87°09′ E, altitude 1630 m). The study area is located in the mid–low mountain zone on the northern slope of the Tianshan Mountains, belonging to a gentle piedmont montane steppe with a temperate continental climate. The soil type is mountain chestnut soil. The annual average temperature is 3.3 °C, annual precipitation is 300 mm, annual evaporation ranges from 1100 to 1300 mm, and the frost-free period lasts 120 to 140 days. The initial soil physicochemical properties of all the experimental plots remained consistent, as listed in Table S2.

The improved grassland in the area was established in 2013. The land was ploughed in that year and then sown with multiple forage species, including *O. viciifolia*, *B. inermis*, *Medicago sativa*, and *Elytrigia repens*. Forages have been harvested by mowing annually ever since, without additional field management measures. At present, *O. viciifolia* and *B. inermis* dominate the grassland.

### 4.2. Research Methods

#### 4.2.1. Experimental Materials

The biochar used in this experiment was purchased from Jiahe Water Purification Materials Co., Ltd., Zhengzhou City, Henan Province, China. It was prepared by air-drying and crushing corn stover, followed by pyrolysis at 300–700 °C. The biochar has a particle size of 200 mesh, specific surface area of 603 m^2^/g, nitrogen content of 1.61%, total carbon content of 70.2%, and ash content of 12.95%.

Urea was selected as the nitrogen fertilizer; it is a white, granular amide nitrogen fertilizer with a nitrogen content of approximately 46% and good water solubility.

#### 4.2.2. Experimental Design

The field experiment was conducted in the improved grassland of Xiejiagou, Urumqi County, in April 2023. A two-factor randomized block design was applied with biochar (B) and nitrogen fertilizer (N) as the two experimental factors. Three biochar rates were arranged: B0 (0 t/hm^2^), B1 (10 t/hm^2^), and B2 (20 t/hm^2^); two nitrogen levels were set: N0 (0 kg/hm^2^) and N15 (225 kg/hm^2^). In total, six combined treatments were formed, namely, B0N0, B0N15, B1N0, B1N15, B2N0, and B2N15, with five replicates for each treatment, resulting in 30 experimental plots in total. Each plot covered an area of 2 m × 4 m, with a 1 m isolation interval between adjacent plots.

Biochar was applied at the end of May 2023. It was evenly spread on the soil surface, mixed into the topsoil with rakes, and sprayed with water for sedimentation and soil adhesion. Biochar was applied only once in the initial experimental year. Urea nitrogen fertilizer was sprayed in 2 to 3 split applications during the growing season every year and fully dissolved in water before use to guarantee a uniform total nitrogen application rate of 225 kg/hm^2^. Forage harvesting was performed three times annually in May, July, and September, with a stubble height of 8 cm, and forage yield was determined simultaneously. No additional field management was implemented throughout the experiment except for the above fertilization, water regulation, and mowing practices (Figure S14).

### 4.3. Field Sampling and Determination Methods

#### 4.3.1. Forage Yield

Forage was mown at the early flowering stage in May, July, and September 2025, with a stubble height of 8 cm. After weighing the fresh forage yield, 200 g fresh samples were randomly collected and oven-dried at 65 °C for 72 h to constant weight. The fresh–dry ratio was calculated to estimate the hay yield.

#### 4.3.2. Leaf Water Content

The leaf water content was determined by the oven-drying method. Leaf samples of *Onobrychis viciifolia* and *Bromus inermis* were collected around the 5th, 15th, and 25th of each month from April to September. The fresh weight was taken first, followed by drying to constant weight to obtain the dry weight. The monthly leaf water content was calculated as the arithmetic mean value of three measurements.

#### 4.3.3. Plant Total Nitrogen

Before each mowing, plant samples of monoculture and mixed-sown sainfoin and smooth brome were collected. All samples were oven-dried at 65 °C, ground, and sieved through a 40-mesh sieve for standby use. Plant total nitrogen content was determined by the Kjeldahl method.

#### 4.3.4. Sampling and Analysis of Soil Physicochemical Properties

After forage mowing in September 2025, soil samples from the 0–20 cm soil layer were collected via the diagonal sampling method using a 5 cm-diameter soil auger. Two soil cores were randomly taken from each plot and fully mixed, then transported to the laboratory in a low-temperature incubator. After removing plant roots and impurities, the samples were sieved through a 2 mm mesh. Part of the fresh soil was stored in a refrigerator at 4 °C for available nitrogen determination, and the rest was naturally air-dried for the analysis of other soil physicochemical indices.

Soil bulk density (BD), soil water content (SWC), soil porosity, and field water-holding capacity were measured by the core cutting ring method. Soil pH was determined with a pH meter at a soil–water ratio of 2.5:1, and electrical conductivity (EC) was measured using a conductivity meter at a soil–water ratio of 5:1. TN was assayed using the Kjeldahl method, while ammonium nitrogen and nitrate nitrogen were determined by a continuous flow analyzer.

#### 4.3.5. Sampling for Soil Water and Nitrogen Dynamics

Soil samples of the 0–10 cm layer were collected with a 3 cm-diameter soil auger on the 1st, 3rd, 7th, 14th, 21st, and 28th day after annual fertilization. Two random soil cores per plot were blended, sieved through a 2 mm mesh, and refrigerated at 4 °C for subsequent available nitrogen analysis.

From April to September, soil samples of the 0–20 cm layer were collected every ten days (around the 5th, 15th, and 25th). Soil moisture content was measured via the oven-drying method at 105 °C to constant weight, and the monthly average water content was calculated from three sample replicates. In addition, soil water contents before forage regreening and after harvest were determined to characterize soil water dynamics throughout the whole growth period.

### 4.4. Data Processing and Statistical Analysis

The experimental data were sorted and summarized using Excel. Statistical analyses were performed using SPSS 27.0 with a significance level of *p* < 0.05, including two-way ANOVA, one-way ANOVA, Duncan’s multiple range test, *t*-test, and Pearson correlation analysis. All data were presented as mean ± standard error, and figures were plotted using Origin 2021.

The partial least squares path model (PLS-PM) was applied to quantify the complex causal relationships between the combined biochar–nitrogen application, soil physicochemical properties, vegetation growth, and economic benefits. The model was established following the logical framework of exogenous regulation → soil physicochemical properties → vegetation growth → economic benefits. The exogenous treatments, soil physicochemical traits, and soil water–nitrogen dynamics were defined as multi-indicator latent variables, while biomass and economic benefits were incorporated as single-indicator observed variables.

All observed indicators were standardized via the Z-score before model construction. Parameter estimation was conducted using the variance-maximization-based partial least squares algorithm, and path significance was verified by bootstrap resampling 500 times. The coefficient of determination (R^2^) and goodness of fit (GoF) were adopted to evaluate the local explanatory power and overall model fitness, respectively. All the above analyses and path topology diagrams were performed in the R 4.5.2 software using the plspm and ggraph packages. The calculation formulas used for other indices are as follows.

Soil available nitrogen content = Soil ammonium nitrogen content + Soil nitrate nitrogen content(1)

Soil water storage (mm) = Soil water content in 0–10 cm layer × Soil bulk density × Soil layer depth(2)

Water-use efficiency [kg/(ha·mm)] = Hay yield/(Initial soil water storage before growth period + Precipitation − Soil water storage at harvest)(3)

Plant nitrogen accumulation (kg/ha) = (Plant nitrogen content rate × Aboveground dry matter yield)/100%(4)

Partial factor productivity of nitrogen fertilizer (kg/kg) = Hay yield under nitrogen application treatment/Nitrogen application rate(5)

Agronomic efficiency of nitrogen fertilizer (kg/kg) = (Hay yield in nitrogen-applied plots − Hay yield in non-nitrogen plots)/Nitrogen application rate(6)

Nitrogen uptake efficiency (kg/ha) = Plant nitrogen accumulation/Nitrogen application rate(7)

Nitrogen-use efficiency (kg/kg) = Biomass (kg/hm^2^)/Plant nitrogen accumulation (kg/hm^2^) (8)

Economic benefit = Average annual forage yield in two years × Unit price of forage − Nitrogen fertilizer cost − Annual average biochar cost(9)

Note: Biochar was applied once in the first year, and the cost of this was averaged over 10 years.

The membership function method in fuzzy mathematics was used to comprehensively evaluate the functional traits of forage under different treatments. The average membership function value of each index under various treatments was calculated using the following formulas:

U(X) = (X − X min)/(X max − X min)(10)

U(X) = (X max − X)/(X max − X min)(11)

## 5. Conclusions

This field investigation, conducted on improved mountain grasslands in arid Xinjiang, elucidated the comprehensive effects and internal regulation mechanisms of biochar combined with nitrogen fertilization. Co-application synchronously optimized soil physical and chemical properties and strengthened soil nitrogen retention, thereby extending the sustained supply of available nitrogen and mitigating nitrogen loss risks. 

The legume *Onobrychis viciifolia* and gramineous *Bromus inermis* exhibited distinct adaptive differences to biochar dosages in terms of water and nitrogen absorption, with their nutrient accumulation decreasing through successive mowing cycles. Moderate biochar input balanced forage productivity and economic profits, while a high biochar dosage reduced net economic returns despite its superiority in long-term soil carbon and nitrogen sequestration. Partial least squares path modeling revealed a dual regulatory framework: combined treatments exerted direct positive effects on the soil water–nitrogen environment, and these improved soil conditions acted as the core mediator in promoting forage biomass and economic benefits via cascading indirect pathways. 

This work provides targeted fertilization strategies and a theoretical basis for the sustainable management of perennial improved grasslands in arid northwest regions of China.

## Figures and Tables

**Table 1:** Preliminary assessment of economic benefits.

Treatment	Average Annual Output (kg/ha)	Income (CNY/ha)	Cost (CNY/ha)	Net Profit (CNY/ha)
B0N0	6964.28	11,630.35	0	11,630.35
B0N15	7193.28	12,012.78	540	11,472.78
B1N0	7435.06	12,416.55	1100	11,316.55
B1N15	8615.56	14,387.99	1640	12,747.99
B2N0	7812.92	13,047.58	2200	10,847.58
B2N15	8197.62	13,690.03	2740	10,950.03

## Data Availability

The data presented in this study are available on request from the corresponding authors. The data are not publicly available due to privacy and ethical restrictions.

## Supplementary Materials

The following supporting information can be downloaded at: https://www.mdpi.com/article/10.3390/plants15142203/s1. Figure S1 Effects of different fertilization methods on soil chemical properties of artificial grassland; Figure S2 Effects of different fertilization methods on soil carbon nitrogen phosphorus stoichiometry in artificial grassland; Figure S3 Effects of different fertilization methods on soil water content of artificial grassland from April to September; Figure S4 Effects of different fertilization methods on soil water content of artificial grassland from April to September; Figure S5 Effects of different fertilization methods on dynamic changes of soil nitrate nitrogen in artificial grassland; Figure S6 Effects of different fertilization methods on dynamic changes of soil ammonium nitrogen in artificial grassland after the third fertilization; Figure S7 Effects of different fertilization methods on leaf water content of *O. viciifolia* from April to September; Figure S8 Effects of different fertilization methods on leaf water content of *B. inermis* from June to July; Figure S9 Effects of different fertilization methods on nitrogen absorption efficiency of *O. viciaefolia*; Figure S10 Effects of different fertilization methods on nitrogen use efficiency of *O. viciaefolia*; Figure S11 Effects of different fertilization methods on nitrogen use efficiency of *B. inermis*; Figure S12 Effects of different fertilization methods on nitrogen use efficiency under different cutting frequencies; Figure S13 Pearson correlation analysis of soil environmental factors and yield of artificial grassland; Figure S14 Summary map of the study area; Table S1 Membership function ranking of soil physical and chemical properties under different treatments; Table S2 Soil basic nutrient information.

## Abbreviations

The following abbreviations are used in this manuscript:

ADF	Acid detergent fiber
ANOVA	Analysis of variance
B0	0 t/hm^2^ biochar
B1	10 t/hm^2^ biochar
B2	20 t/hm^2^ biochar
B0N0	Diammonium phosphate
B0N15	Dry matter digestibility
B1N0	Dry-to-fresh ratio
B1N15	10 t/hm^2^ biochar plus 225 kg/hm^2^ nitrogen fertilizer
B2N0	Dry matter
B2N15	20 t/hm^2^ biochar plus 225 kg/hm^2^ nitrogen fertilizer
BD	Soil bulk density
CLL	Compound leaf length
CLN	Compound leaf number
C/N	Carbon–nitrogen ratio
EC	Electrical conductivity
GoF	Global goodness-of-fit
IL	Internode length
IN	Internode number
LN	Leaflet number
LL	Leaf length
LW	Leaf width
MDA	Malondialdehyde
N0	0 kg/hm^2^ nitrogen
N15	225 kg/hm^2^ nitrogen
Nb	Leaf number
PH	Plant height
PLS-PM	Partial least squares path model
Pro	Proline
RGR	Relative growth rate
RL	Root-related indicators of taproot length
R/S	Root–shoot ratio
SD	Stem diameter
SEM	Structural equation model
SOD	Superoxide dismutase
SS	Soluble sugar
SP	Soluble protein
SWC	Soil water content
TBA	Thiobarbituric acid
TC	Total carbon
TF	Total flavonoids
TN	Total nitrogen
WUE	Water-use efficiency
