# Supplementary material for: Effects of Combined Application of Biochar and Nitrogen Fertilizer on Forage Growth and Water- and Nitrogen-Use Efficiency in Managed Grassland
Source: Plants (Basel). 2026 Jul 19;15(14):2203. doi: 10.3390/plants15142203 (PMC13417073; doi:10.3390/plants15142203)
Supplement: Supplementary file 1 [file plants-15-02203-s001.zip › plants-4363035-supplementary.pdf]

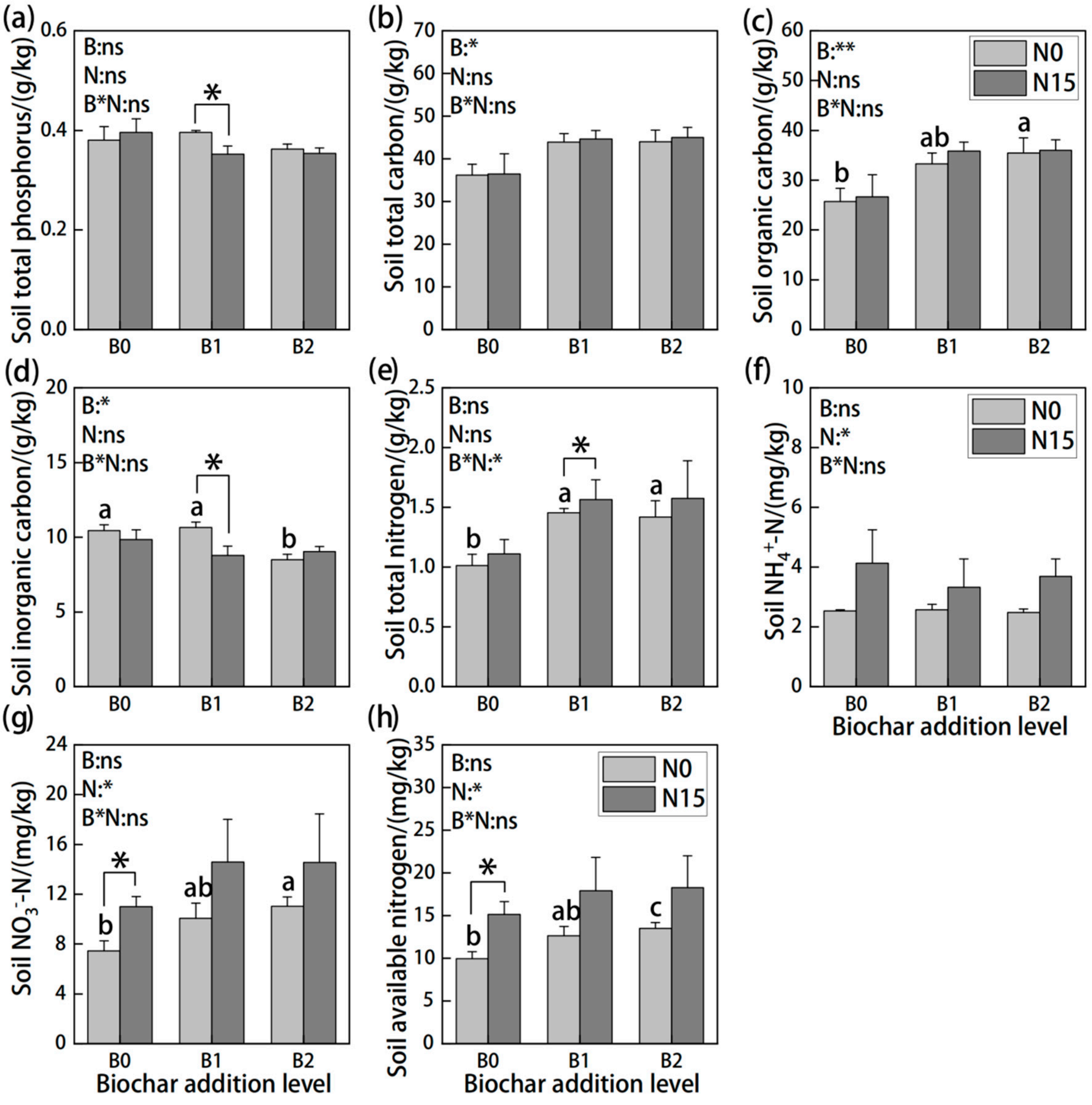


Figure S1 Effects of different fertilization methods on soil chemical properties of artificial grassland

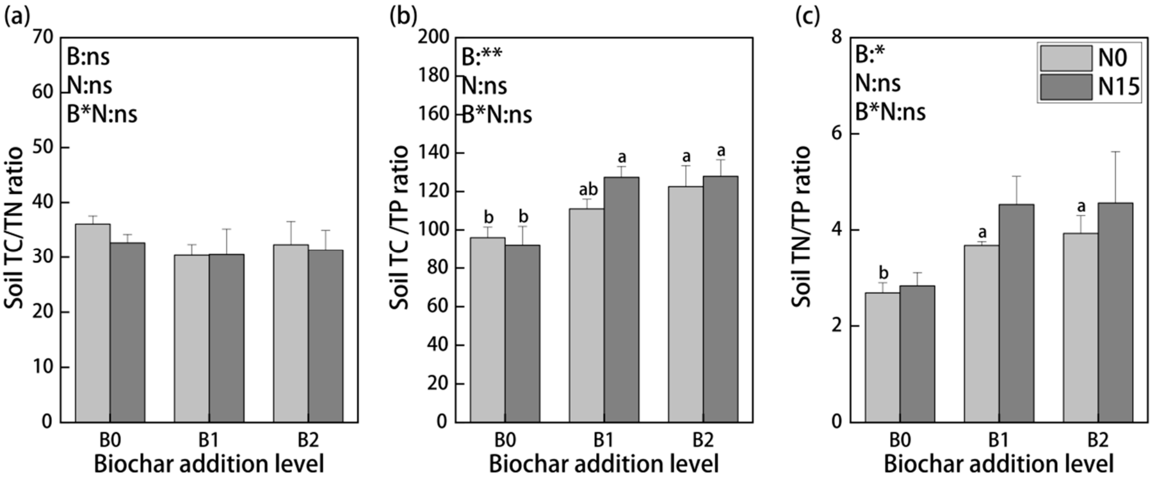

Figure S2 Effects of different fertilization methods on soil carbon nitrogen phosphorus stoichiometry in artificial grassland

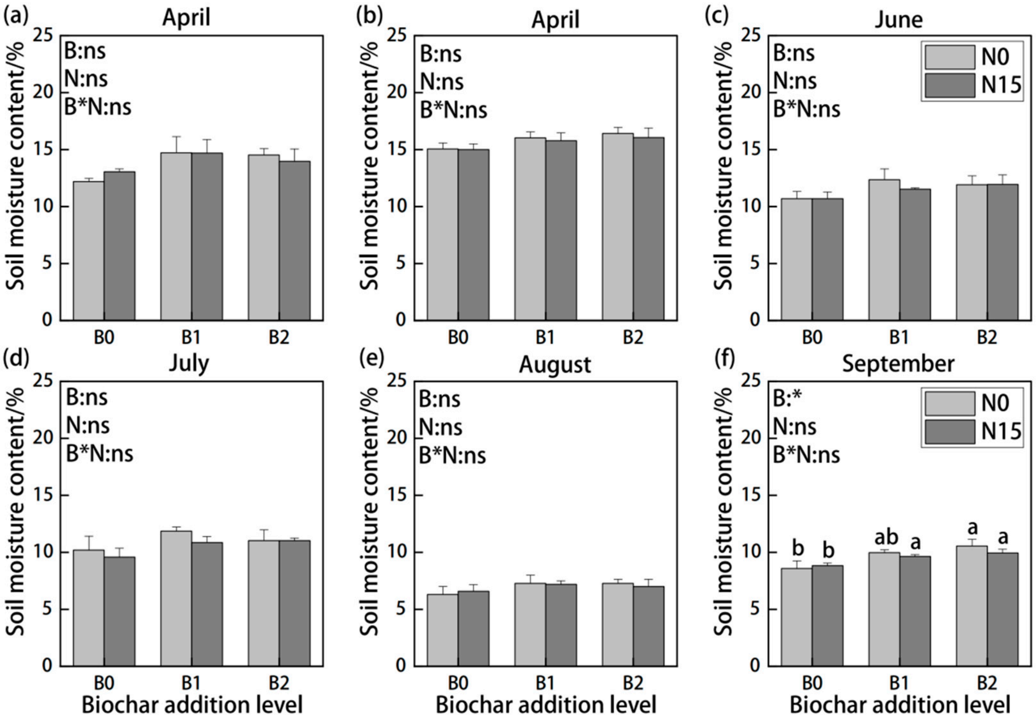


Figure S3 Effects of different fertilization methods on soil water content of artificial grassland from April to September

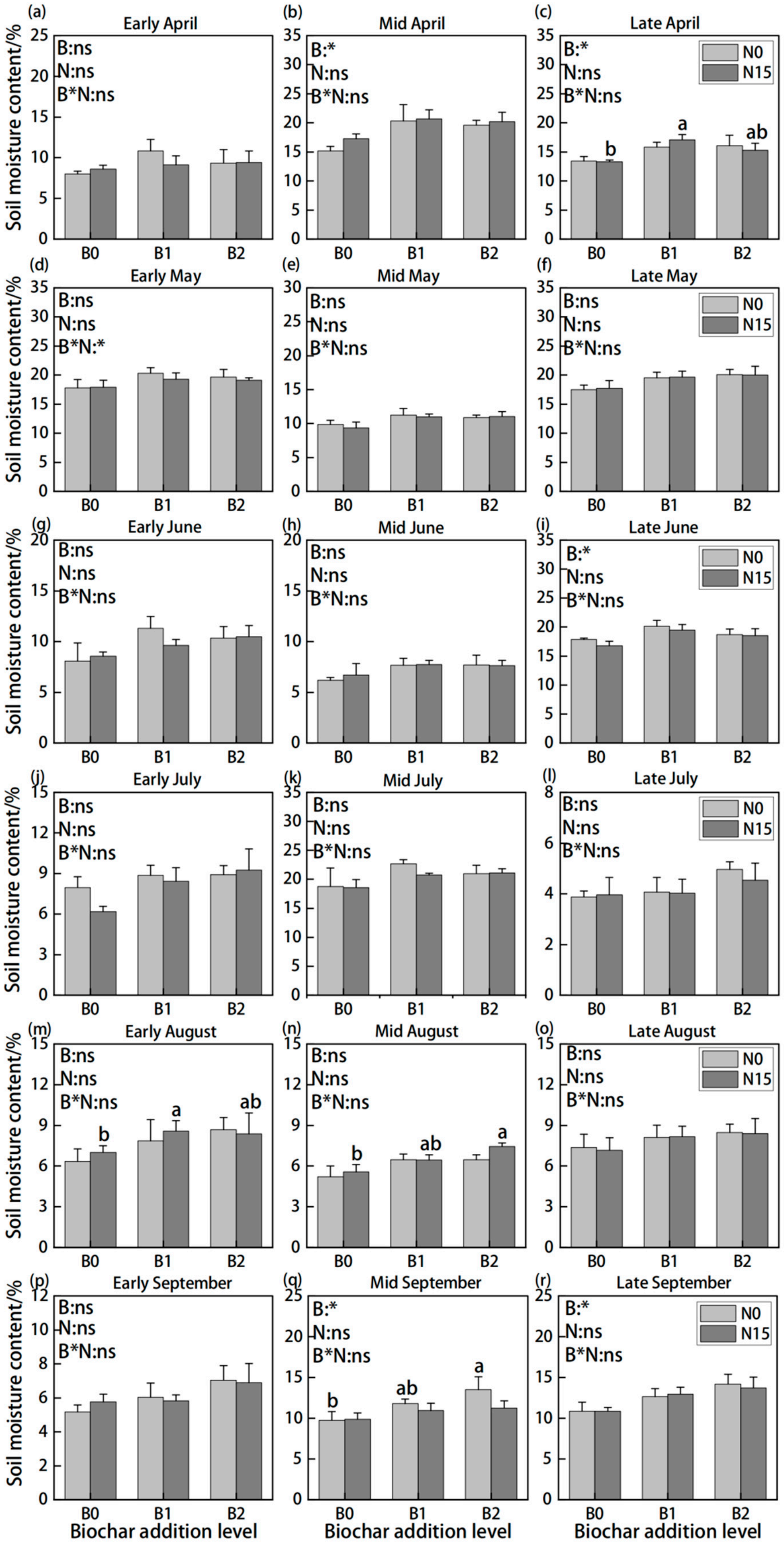


Figure S4 Effects of different fertilization methods on soil water content of artificial grassland from April to September

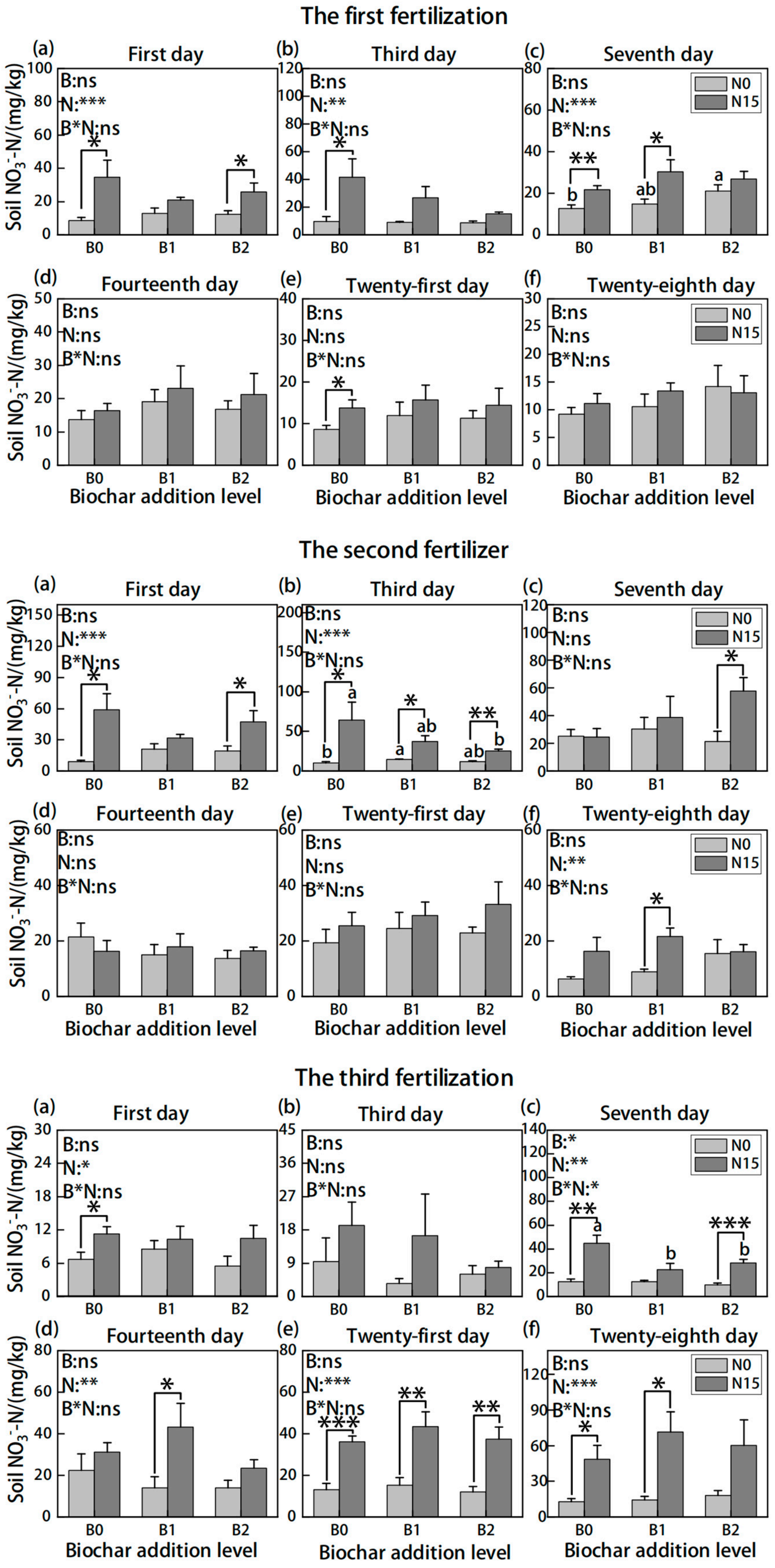


Figure S5 Effects of different fertilization methods on dynamic changes of soil nitrate nitrogen in

artificial grassland

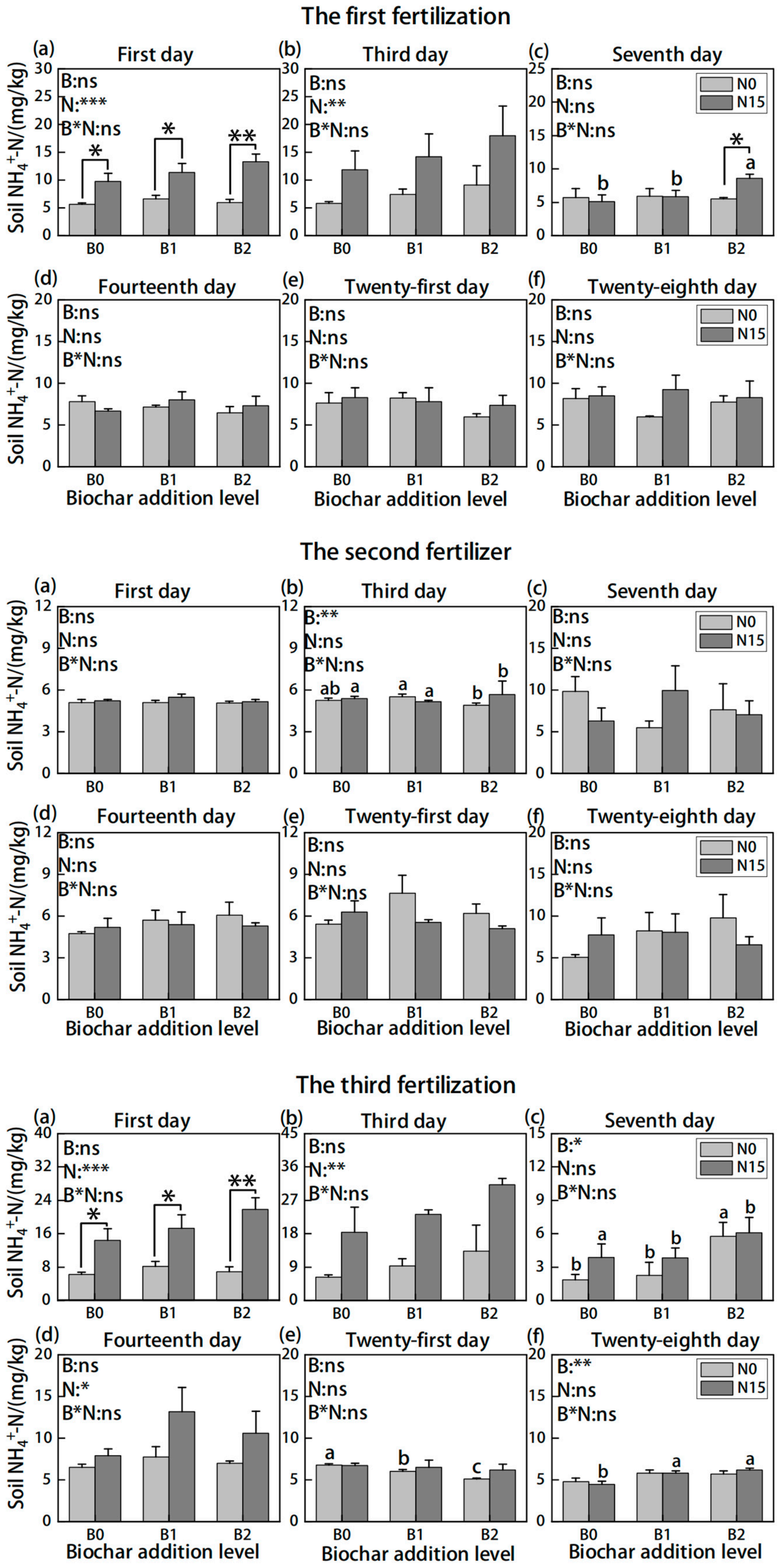

Figure S6 Effects of different fertilization methods on dynamic changes of soil ammonium nitrogen in artificial grassland after the third fertilization

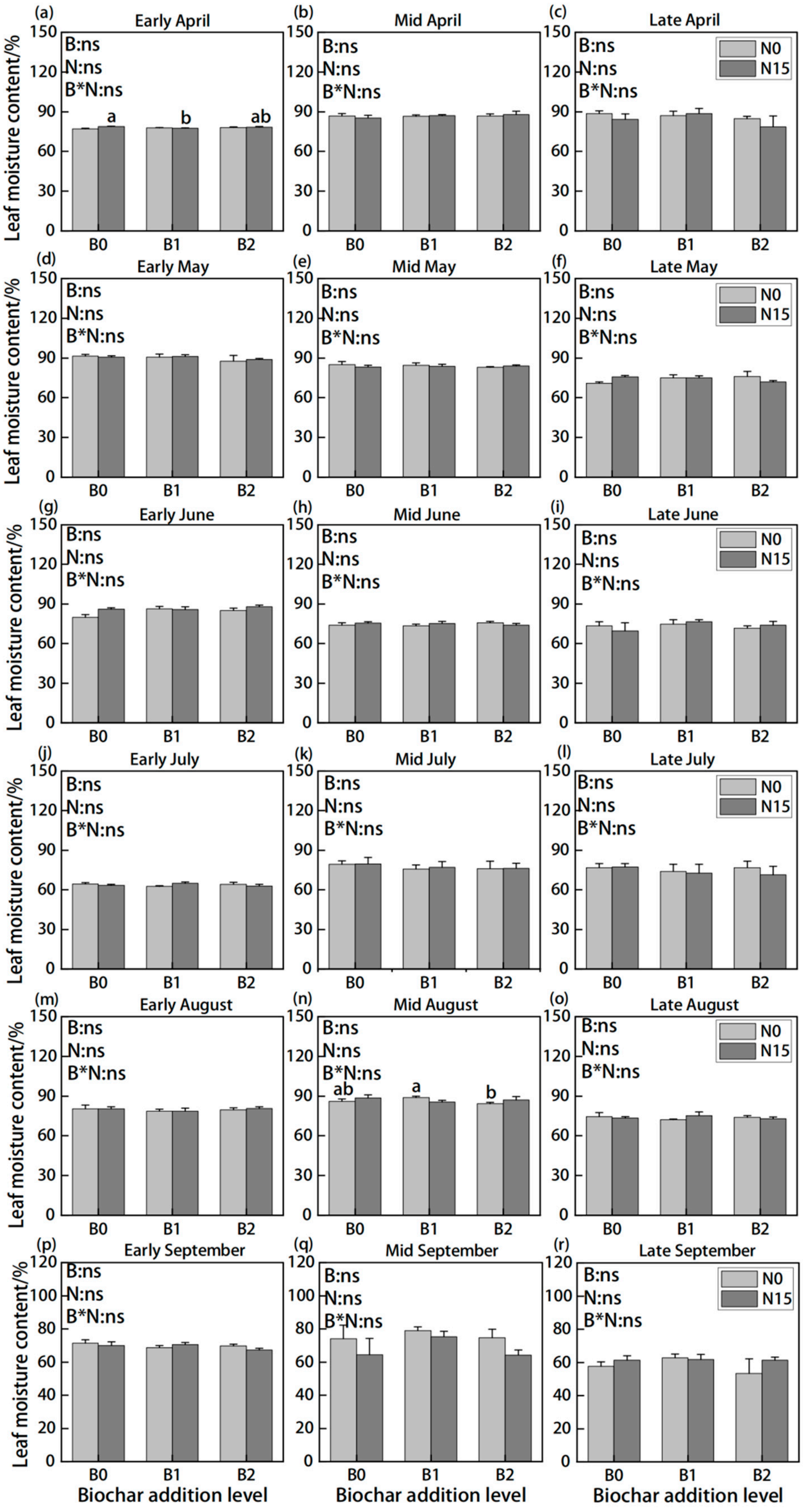

Figure S7 Effects of different fertilization methods on leaf water content of *O. viciifolia* from April to September

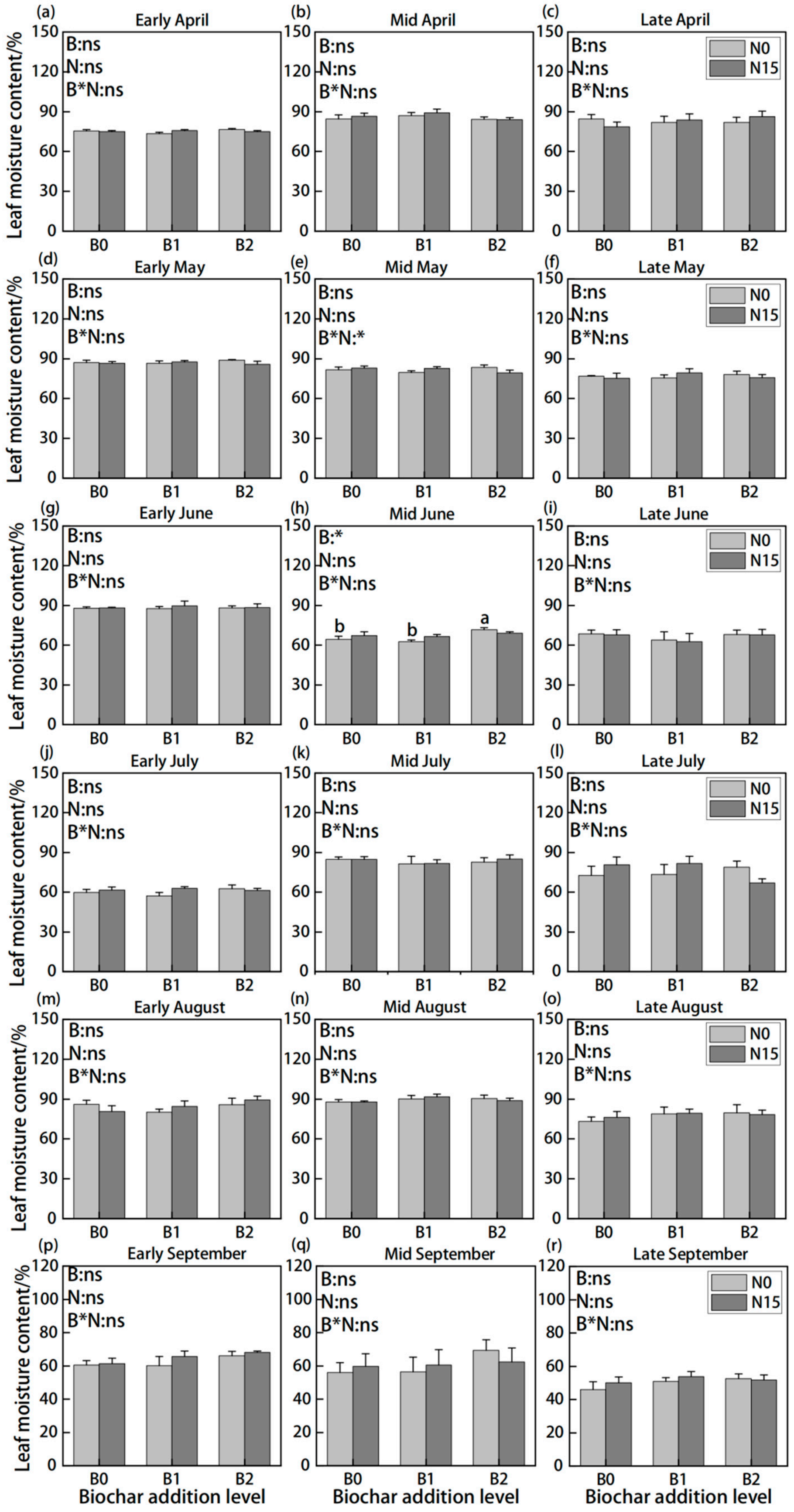

Figure S8 Effects of different fertilization methods on leaf water content of *B. inermis* from June to July

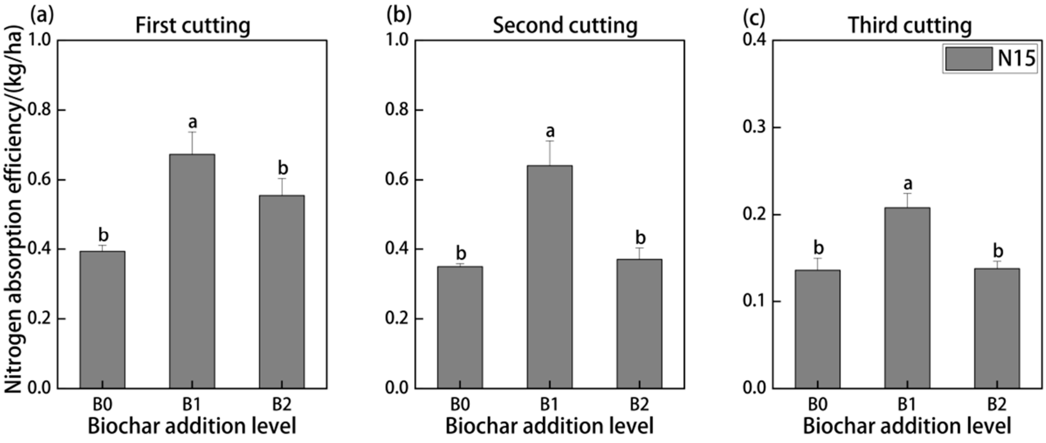


Figure S9 Effects of different fertilization methods on nitrogen absorption efficiency of *O. viciaefolia*

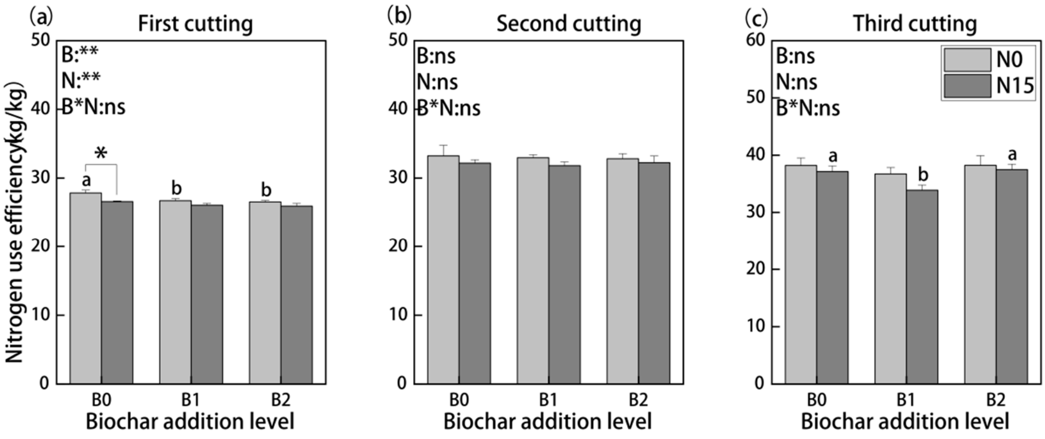


Figure S10 Effects of different fertilization methods on nitrogen use efficiency of *O. viciaefolia*

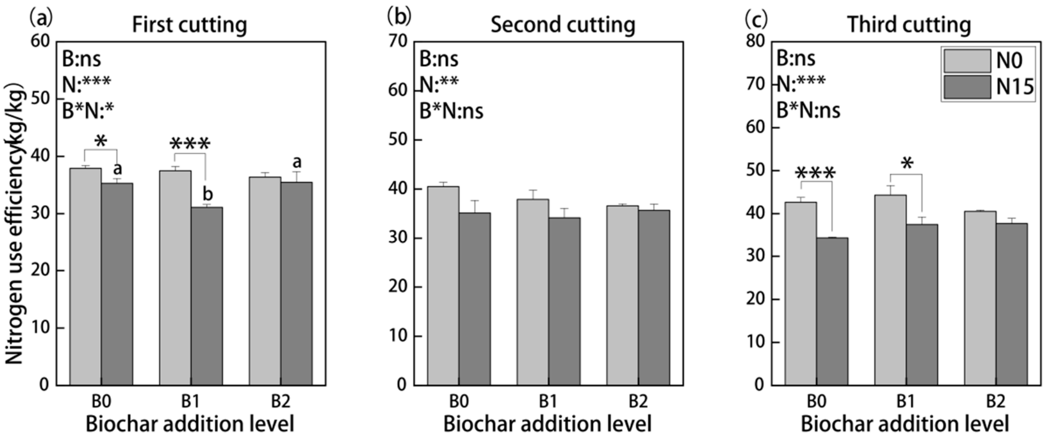


Figure S11 Effects of different fertilization methods on nitrogen use efficiency of *B. inermis*

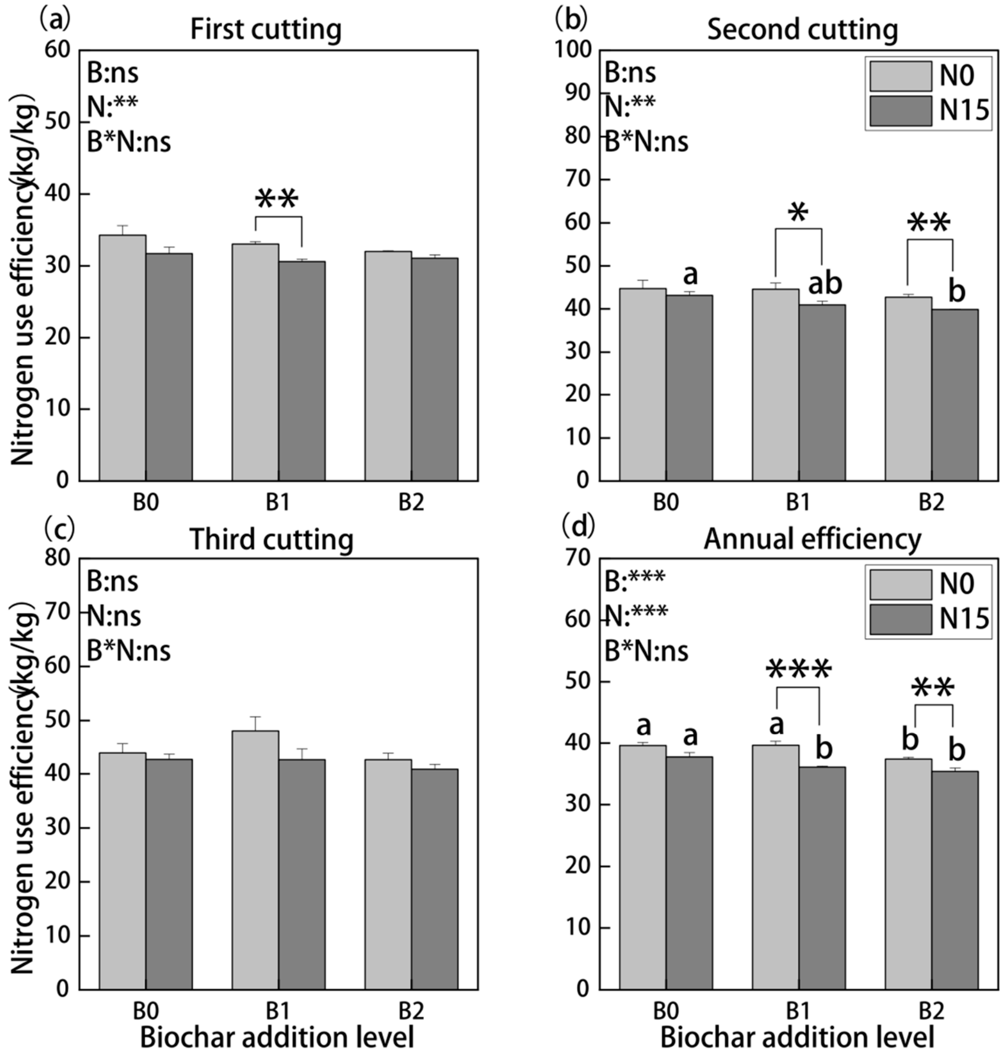


Figure S12 Effects of different fertilization methods on nitrogen use efficiency under different cutting frequencies

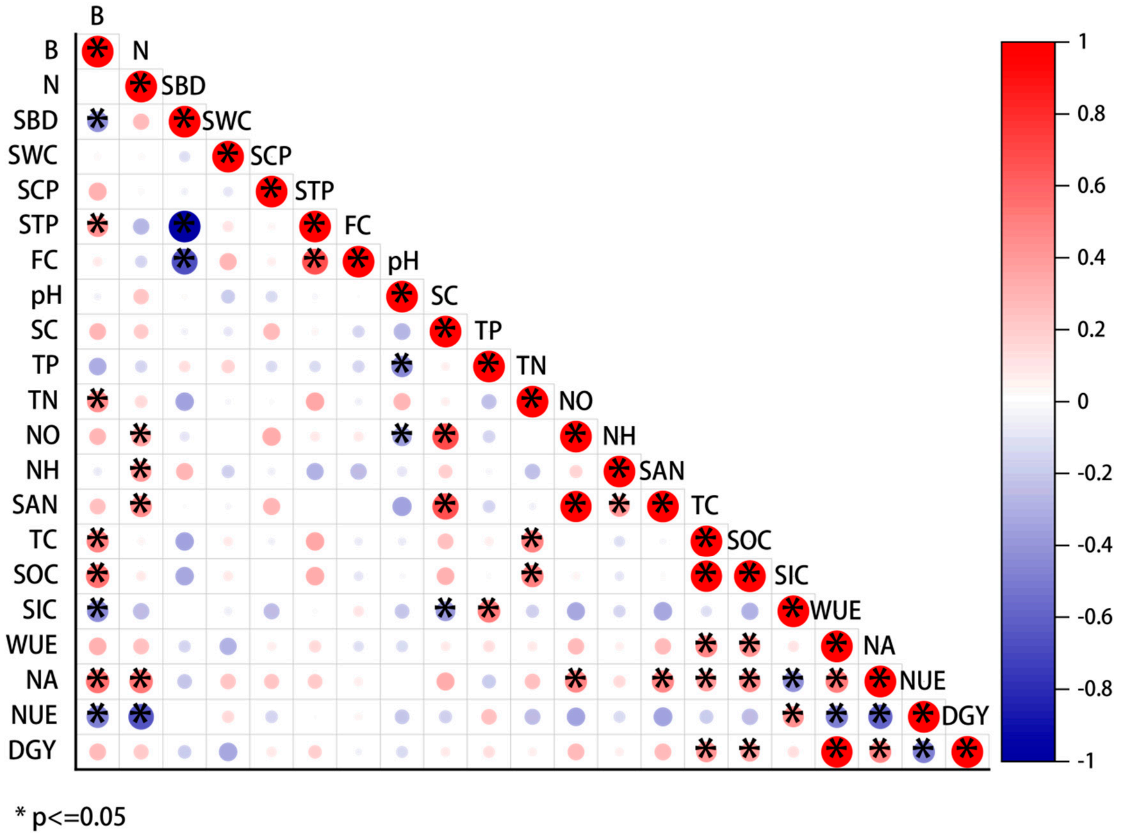


Figure S13 Pearson correlation analysis of soil environmental factors and yield of artificial grassland

Table S1 Membership function ranking of soil physical and chemical properties under different treatments

| Index | B0N0 | B0N15 | B1N0 | B1N15 | B2N0 | B2N15 |
|---|---|---|---|---|---|---|
| Soil bulk density | 0.090 | 0.000 | 1.000 | 0.452 | 0.505 | 0.471 |
| Soil moisture content | 0.134 | 0.000 | 0.319 | 0.238 | 0.243 | 1.000 |
| Soil capillary porosity | 0.000 | 0.128 | 1.000 | 0.217 | 0.222 | 0.217 |
| Soil total porosity | 0.000 | 0.047 | 0.299 | 0.128 | 0.661 | 1.000 |
| Soil field capacity | 0.089 | 0.000 | 1.000 | 0.451 | 0.505 | 0.474 |
| Soil pH | 0.854 | 0.793 | 0.854 | 0.000 | 1.000 | 0.817 |
| Soil conductivity | 0.000 | 0.218 | 0.364 | 0.831 | 0.521 | 1.000 |
| Soil total phosphorus | 0.364 | 0.000 | 0.000 | 1.000 | 0.773 | 0.955 |
| Soil total nitrogen | 0.000 | 0.174 | 0.786 | 0.979 | 0.722 | 1.000 |
| Soil $NO_3^-$-N | 0.000 | 0.498 | 0.367 | 1.000 | 0.502 | 0.997 |
| Soil $NH_4^+$-N | 0.024 | 1.000 | 0.051 | 0.513 | 0.000 | 0.733 |
| Soil available nitrogen | 0.000 | 0.623 | 0.321 | 0.959 | 0.428 | 1.000 |
| Soil total carbon | 0.000 | 0.034 | 0.875 | 0.956 | 0.879 | 1.000 |

| | | | | | | |
|---|---|---|---|---|---|---|
| Soil organic carbon | 0.000 | 0.088 | 0.735 | 0.986 | 0.947 | 1.000 |
| Soil inorganic carbon | 0.090 | 0.372 | 0.000 | 0.869 | 1.000 | 0.756 |
| Soil TC/TN ratio | 0.000 | 0.601 | 1.000 | 0.981 | 0.661 | 0.834 |
| Soil TC/TP ratio | 0.110 | 0.000 | 0.528 | 0.986 | 0.851 | 1.000 |
| Soil TN/TP ratio | 0.000 | 0.077 | 0.528 | 0.981 | 0.662 | 1.000 |
| annual water use efficiency | 0.000 | 0.121 | 0.232 | 1.000 | 0.494 | 0.734 |
| annual nitrogen accumulation | 0.000 | 0.243 | 0.193 | 1.000 | 0.523 | 0.907 |
| annual nitrogen use efficiency | 0.988 | 0.554 | 1.000 | 0.159 | 0.472 | 0.000 |
| annual hay yield | 0.000 | 0.138 | 0.285 | 1.000 | 0.513 | 0.747 |
| Average | 0.125 | 0.259 | 0.534 | 0.713 | 0.595 | 0.801 |
| Ranking | 6 | 5 | 4 | 2 | 3 | 1 |

Table S2 Soil basic nutrient information

| Soil pH | Soil electric conductivity (μS/cm) | Soil bulk density($g/cm^3$) | Soil water content(%) | Total carbon (g/kg) | Total nitrogen (g/kg) | Total phosphorus (g/kg) |
|---|---|---|---|---|---|---|
| 8.08 ± 0.9 | 125.48 ± 30.35 | 1.51 ± 0.70 | 9.63 ± 0.93 | 36.19 ± 7.70 | 1.01 ± 0.32 | 0.36 ± 0.2 |

Note: Data is expressed as mean ± standard error.

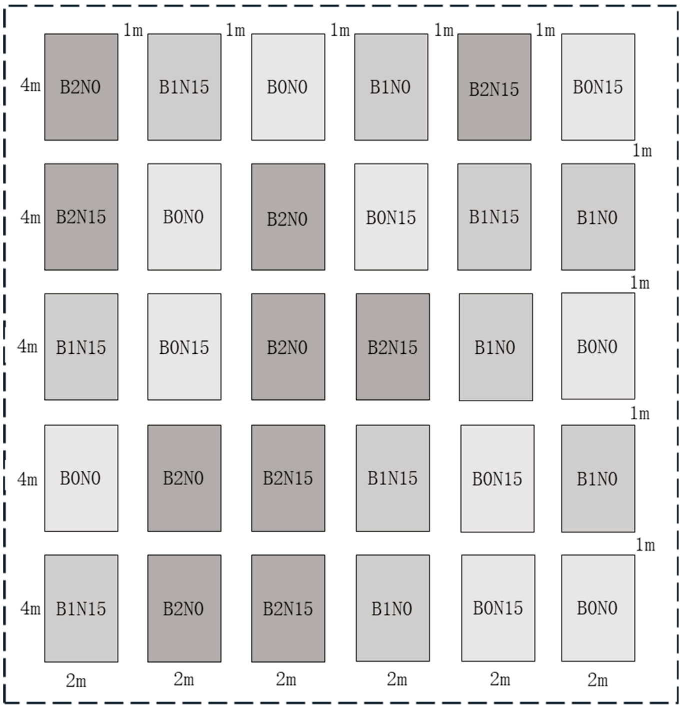


Figure S14 Summary map of the study area
